# Bispecific BCMA-CD3 Antibodies Block Multiple Myeloma Tumor Growth

**DOI:** 10.3390/cancers14102518

**Published:** 2022-05-20

**Authors:** Lijun Wu, Yanwei Huang, John Sienkiewicz, Jinying Sun, Liselle Guiang, Feng Li, Liming Yang, Vita Golubovskaya

**Affiliations:** 1 Promab Biotechnologies, 2600 Hilltop Drive, Richmond, CA 94806, USA; john@promab.com (L.W.); yanwei.huang@promab.com (Y.H.); john.sienkiewicz@promab.com (J.S.); sunnie.sun@promab.com (J.S.); liselle.guiang@promab.com (L.G.); feng.li@promab.com (F.L.); liming.yang@promab.com (L.Y.); 2 Forevertek Biotechnology, Janshan Road, Changsha Hi-Tech Industrial Development Zone, Changsha 410205, China

**Keywords:** multiple myeloma, BCMA, bispecific antibody

## Abstract

**Simple Summary:**

Multiple myeloma accounts for approximately 10% of hematological cancers in the United States. It is a malignancy of plasma cells which accumulate in the bone marrow and produce a monoclonal protein. Novel treatments are needed to cure multiple myeloma patients. The goal of this report was to develop novel bispecific BCMA-CD3 antibodies targeting a B cell maturation antigen, BCMA, which is overexpressed in multiple myeloma. The data demonstrated high efficacy of BCMA-CD3 antibodies in multiple myeloma cell lines in vitro and in vivo. The data provide a basis for future clinical studies.

**Abstract:**

BCMA antigen is overexpressed in multiple myeloma cells and has been shown to be a promising target for novel cellular and antibody therapeutics. The humanized BCMA (clone 4C8A) antibody that effectively targeted multiple myeloma in a CAR (chimeric antigen receptor) format was used for designing several formats of bispecific BCMA-CD3 antibodies. Several different designs of univalent and bivalent humanized BCMA-CD3 CrossMAB and BCMA-FAB-CD3 ScFv-Fc antibodies were tested for binding with BCMA-positive cells and T cells and for killing by real time cytotoxic activity and IFN-gamma secretion with CHO-BCMA target cells and with multiple myeloma MM1S and H929 cell lines. All BCMA-CD3 antibodies demonstrated specific binding by FACS to CHO-BCMA, multiple myeloma cells, and to T cells with affinity Kd in the nM range. All antibodies with T cells specifically killed CHO-BCMA and multiple myeloma cells in a dose-dependent manner. The BCMA-CD3 antibodies with T cells secreted IFN-gamma with EC_50_ in the nM range. In addition, three BCMA bispecific antibodies had high in vivo efficacy using an MM1S xenograft NSG mouse model. The data demonstrate the high efficacy of novel hBCMA-CD3 antibodies with multiple myeloma cells and provide a basis for future pre-clinical and clinical development.

## 1. Introduction

Multiple myeloma accounts for 1.3% of all cancers and more than 10% of hematological diseases in the USA [1,2]. Multiple myeloma (MM) is characterized by clonal expansion of plasma cells in the bone marrow resulting in the expression of myeloma M protein (also called monoclonal protein) and causing different organ damage [1]. Although current therapies for multiple myeloma cause remission, most of the patients will eventually die due to relapse [3]. Novel immunotherapy treatments are needed for multiple myeloma with novel monoclonal antibodies, antibody drug conjugates, ADC, CAR (chimeric antigen receptor)-T cell therapy, or bispecific antibodies targeting specific multiple myeloma antigens [3,4,5,6,7,8,9,10,11,12,13].

BCMA (CD269 or tumor necrosis factor receptor superfamily member 17 (TNFRSF17)) is a B cell maturation antigen that is overexpressed in malignant plasma B cells [14]. BCMA is one of the multiple myeloma antigens which has emerged as a promising therapeutic target [6,15,16,17]. BCMA binds several ligands including APRIL (a proliferation-inducing ligand) and BAFF (B cell-activating factor) [18,19] and plays an important role in survival signaling mediated by NF-kappa B, STAT3, ERK1/2, and AKT/PI3K signaling pathways [13,14,19,20].

In this report, we generated novel BCMA-CD3 antibodies using several different designs and tested their efficacy in vitro and in vivo. The data demonstrate the functional activity of novel BCMA-CD3 bispecific antibodies which can be used in future pre-clinical and clinical studies.

## 2. Materials and Methods

### 2.1. Cell Lines

Multiple myeloma H929, RPMI8226, and MM1S cell lines and lymphoblast K562 cell lines were purchased from the ATCC (Manassas, VA, USA) and cultured in RPMI-1640 medium (Thermo Fisher, Waltham, MA, USA) with 10% FBA (AmCell, Mountain View, CA, USA). CHO cells were cultured in DMEM (GE Healthcare, Chicago, IL, USA) containing 10% FBS (AmCell, Mountain View, CA, USA). CHO-BCMA cells were purchased from BPS Bioscience (San Diego, CA, USA) and cultured in Ham’s F12K medium containing 10% FBS with 1 mg/mL Geneticin (Thermo Fisher). Human peripheral blood mononuclear cells (PBMC) were isolated from whole blood at the Stanford Hospital Blood Center according to IRB-approved protocol (#13942). PBMC were isolated by density sedimentation over Ficoll-Paque (GE Healthcare). B cells were isolated from PBMC using a human B cell isolation kit according to the manufacturer’s protocol (Myltenyi Biotech, Bergisch Gladbach, Germany). B cells were expanded for 7 days using IMDM (Iscove’s Modified Dulbecco’s Medium) with 10% human AB serum, IL-21 (50 ng/mL), CD40 ligand (100 ng/mL), and anti-HA antibody (200 ng/mL). B cell marker was detected on B cells by FACS with an anti-CD20 antibody.

### 2.2. Cloning of Bispecific Antibodies 

Different designs of BCMA CrossMab KIH IgG1 bivalent and univalent were constructed according to [21]. The Fc region contained P329G mutation and L234AL235A (LALA) mutations to silence Fc by preventing binding to Fc gamma receptors and activating innate immune cells [21]. Humanized BCMA VH and VL from antibody 4C8A clones were used for bispecific antibody generation [22,23]. CD3 Scfv and VH and VL humanized sequences were from CD3e antibodies [21]. Another design was triple chain with BCMA univalent part and CD3 ScFv with KIH and LALA mutations. For one of triple chain designs antibody differently humanized CD3 e was used. All three or four subunits of bispecific antibody were subcloned into pYD11 vector and DNA constructs used for co-transfection of 293 cells at equal ratio. The four subunits of PF3135 (PF-06863135) bispecific BCMA-CD3 antibody sequences were obtained from https://drugs.ncats.io/drug/L0HR9A577V (accessed on 18 May 2022) and each subunit was subcloned into PYD11 vector and engineered antibody (PBM0057) was used as a positive control. The sequences of all antibodies are shown in Supplemental Table S1.

### 2.3. Transfection of 293 Cells, Isolation and Purification of Bispecific Antibodies

293S cells were transfected using DNA constructs of bispecific antibodies and NanoFect transfection agent and incubated in Freestyle F17 medium with 8 mM Glutamine and 0.1% Pluoronic F68 surfactant in suspension bottles using shaker at 37 °C and 5% CO_2_. The supernatant was collected at days 3–7 for antibody purification by centrifugation for 12 min at 3000× *g*. The filtered supernatant was used for the isolation of antibody using column with protein A beads (Millipore, Burlington, MA, USA). After washing column with 1× PBS, the antibody was eluted with Pierce IgG elution buffer (ThermoFisher). The concentration of bispecific antibody was estimated with Nanodrop instrument and BCA protein assay kit (Thermo Fisher). The purified antibodies were run on SDS gel and used for functional analyses. 

### 2.4. Flow Cytometry

To measure binding of bispecific antibodies 0.25 million cells were suspended in 100 µL of buffer (PBS containing 2 mM EDTA pH 8 and 0.5% BSA) and incubated on ice with 1 µL of human serum (Jackson Immunoresearch, West Grove, PA, USA) for 10 min. Then different dilutions of antibody were added, and the cells were incubated on ice for 30 min. The cells were rinsed with FACS buffer and suspended in 100 µL of buffer. Then 1 µL of phycoerythrin (PE) or APC-conjugated secondary antibody (BD Biosciences, San Jose, CA, USA) was added, and the cells were incubated on ice for 30 min. The cells were rinsed and suspended in buffer for analysis on a FACSCalibur (BD Biosciences). Affinity (Kd) was measured with GraphPad software. CAR-positive cells were detected by FACS with anti-mouse F(ab)’2 antibody and biotin-conjugated recombinant BCMA protein added with CD3-APC-conjugated mouse anti-CD3 antibody and PE-conjugated streptavidin at 1:100 dilution. After 30 min incubation at 4 °C, the cells were rinsed, stained with 7-AAD, suspended in the FACS buffer, and analyzed on a FACSCalibur (BD Biosciences).

### 2.5. Real-Time Cytotoxicity Assay (RTCA)

Adherent target cells (CHO or CHO-BCMA) were seeded into 96-well E-plates (Acea Biosciences, San Diego, CA, USA) at 1 × 10^4^ cells per well and monitored in culture overnight with the impedance-based real-time cell analysis (RTCA) × CELLigence system (Acea Biosciences). The next day, the medium was removed and replaced with AIM V-AlbuMAX medium containing 10% FBS ± 1 × 10^5^ effector cells (CAR-T cells, non-transduced T cells, or T cells with BCMA-CD3 bispecific antibodies), in triplicate. The cells in the E-plates were monitored for another one to two days with the RTCA system, and impedance was plotted over time. 

### 2.6. ELISA (Enzyme-Linked Immunoassay)

Target cells (H929, RPMI8226, and MM1S) were cultured with different dilutions of BCMA-CD3 antibodies in U-bottom 96-well plates with 200 µL of AIM V-AlbuMAX medium containing 10% FBS, in triplicate and then analyzed by ELISA for human IFN-γ levels using a kit from R&D Systems (Minneapolis, MN, USA) according to the manufacturer’s protocol. For adherent CHO and CHO-BCMA supernatant after cytotoxicity assay was collected and analyzed as above by ELISA. 

### 2.7. Lentiviral CAR and CAR-T Cell Generation

Lentiviral CAR construct containing humanized BCMA ScFv with same VH and VL as used in BCMA-CD3 bispecific antibodies, 41BB costimulatory and CD3 activating domains was ordered from Vector Builder (Chicago, IL, USA). Lentiviral CAR was used for lentivirus generation as described in [22]. In brief, CAR-T cells were generated by transducing lentiviral CAR into PBMC pre-activated with CD3/CD28 Dynabeads from Thermo Fisher as described in [22]. CAR-T cells were expanded over 10–12 days using AIM-AlbuMAX medium (Thermo Fisher, USA) with 10% FBS and 10 ng/mL IL-2 (Thermo Fisher). 

### 2.8. Mouse RPMI8226 Xenograft Tumors

Six-week-old NSG mice (Jackson Laboratories, Bar Harbor, ME, USA) were housed and manipulated in strict accordance with the Institutional Animal Care and Use Committee (IACUC) (#LUM-001). Each mouse was injected intravenously on day 0 with 100 µL of 2 × 10^6^ MM1S-luciferase positive cells and 5 × 10^6^ PBMC. Bispecific antibodies were injected intravenously (50 ug/mice) on days 4 and 11, and (100 ug/mice) on day 18 as described in [21]. A total of 2 × 10^6^ RPMI8226-luciferase^+^ cells were injected intravenously to NSG mice, and next day 1 × 10^7^ CAR-T cells were injected intravenously to perform imaging and survival study. Imaging was performed using IVIS Imaging System (Perkin Elmer, Watham, MA, USA), and quantification of BLI (bioluminescence) in photons/sec was used for the analysis of xenograft tumor growth. Kaplan Myer curve was used for analysis of mice survival.

### 2.9. Statistical Analysis

Data were analyzed and plotted with Prism software (GraphPad, San Diego, CA, USA). Comparisons between three or more groups were performed by one-way ANOVA or two-way ANOVA with Tukey’s or Sidak’s post hoc test. Comparisons between two groups were performed by unpaired Student’s *t*-test. The survival curves were compared with Log-rank Mantel-Cox test. The difference with *p* < 0.05 was considered significant, and with *p* < 0.001 was considered highly significant.

## 3. Results

### 3.1. Structures of Bispecific BCMA-CD3 Antibodies

**Figure 1:**
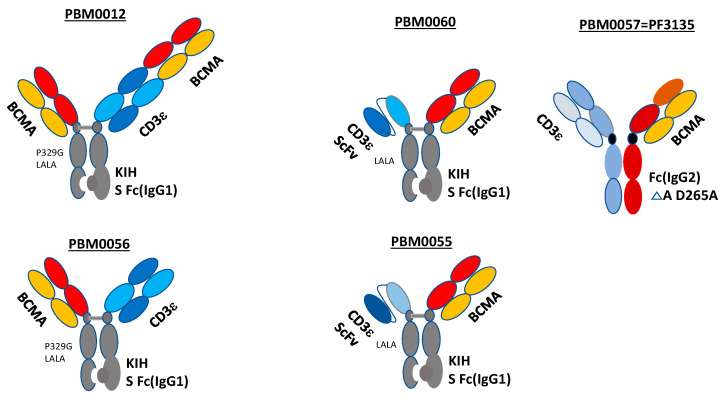
Different structures of BCMA-CD3 antibodies. Left panels: Bivalent PBM0012 antibody Crossmab KIH (**upper panel**) and univalent PBM0056 BCMA-CD3 CrossMAB KIH (IgG1) (**lower panel**) antibodies. Middle upper panel: BCMA-FAB-CD3 Scfv KIH, called PBM0060 (CD3 ScFv contains same heavy and light chains as for PBM0012 and PBM0056); middle lower panel: PBM0055 structure is the same as for PBM0060 antibody but with differently humanized CD3 ScFv. Right upper panel: Pfizer PF3135 control benchmark antibody, called PBM0057.

We designed BCMA-CD3 antibodies with different structures such as univalent, bivalent BCMA-CD3 CrossMab knob-in-hole KIH and BCMA-CD3 ScFv triple chain antibodies (Figure 1). Bivalent CrossMAB and knob-in-hole KIH (IgG1) BCMA-CD3 antibodies are shown on the left upper panel, called PBM0012. The Fc part of BCMA-CD3 antibody contains modification Pro329Gly, called P329G, and Leu234Ala/Leu235Ala, called LALA mutations, as described in [21] (Figure 1). These mutations decrease the binding of BCMA-CD3 antibody to Fc gamma receptors and to complement components preventing Fc gamma receptor-mediated activation of innate immune effector cells including natural killer (NK) cells, monocytes/macrophages, and neutrophils [21]. The same structure but univalent BCMA-CD3 antibody is shown on the lower-left panel and called, PBM0056. The BCMA-CD3 antibody with univalent BCMA arm and CD3 ScFv and with KIH Fc with LALA mutations is shown in Figure 1, middle-upper panel, called PBM0060. The same structure but with a differently humanized CD3 ScFv sequence with KIH Fc with LALA mutations is shown in Figure 1, middle-lower panel, called PBM0055. The control benchmark antibody was Pfizer’s PF3135 antibody with IgG2 Fc with D265A mutation, called PBM0057 (Figure 1, upper right panel).

### 3.2. Binding of BCMA-CD3 Antibodies to BCMA-Positive Cells and T Cells

**Figure 2:**
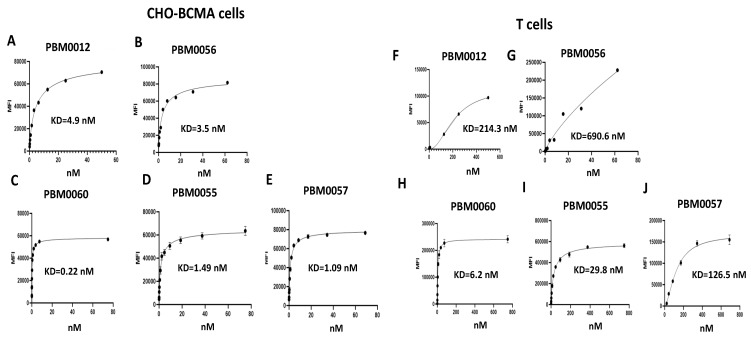
Binding of BCMA-CD3 antibodies to BCMA-positive CHO-BCMA cells and T cells. FACS was performed with all antibodies on BCMA-positive CHO-BCMA cells (**A**–**E**), and T cells (**F**–**J**). Representative assay is shown.

All formats of BCMA-CD3 antibodies shown in Figure 1 were tested with CHO-BCMA-positive cells by FACS for binding (Figure 2A–E). All antibodies bound CHO-BCMA cells at Kd in nM range from 0.22–4.9 nM. There was no binding of BCMA-CD3 antibodies to CHO cells (Figure S1A). These antibodies also bound to CD3-positive T cells with KD that varied from 6.2 nM to 690 nM (Figure 2F–J). Thus, BCMA-CD3 antibodies bound both BCMA-positive and CD3-positive cells.

### 3.3. T Cells with BCMA-CD3 Antibody Kill CHO-BCMA-Positive Cells and Secrete IFN-Gamma

**Figure 3:**
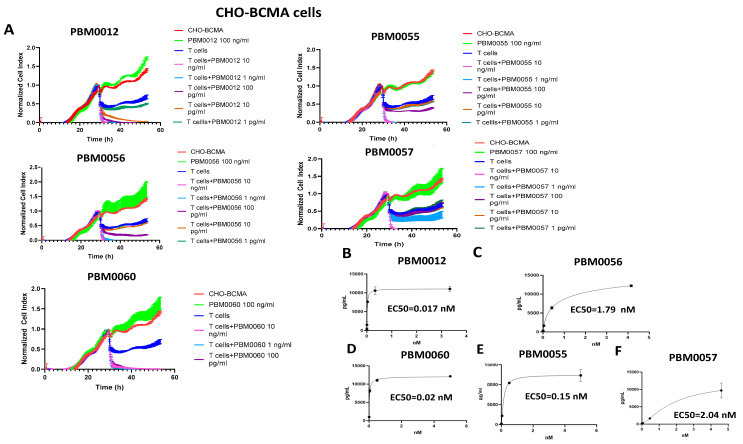
T cells with BCMA-CD3 antibodies effectively killed CHO-BCMA cells and secreted IFN-gamma. (**A**) RTCA killing assay with T cells and BCMA-CD3 antibodies against CHO-BCMA target cells. (**B**–**F**) IFN-gamma secretion by T cells with BCMA-CD3 antibodies and CHO-BCMA target cells. EC50 for each antibody is shown in nM. Representative RTCA and IFN-gamma assays are shown.

T cells with different dilutions of BCMA-CD3 antibodies were used in a real-time cytotoxicity assay (RTCA) with CHO-BCMA target cells (Figure 3A). All designs of BCMA-CD3 antibodies with T cells killed CHO-BCMA in a dose-dependent manner (Figure 3A). BCMA-CD3 antibody alone without T cells did not have cytotoxic activity. 

BCMA-CD3 bispecific antibodies with T cells secreted IFN-gamma with EC50 ranged from 0.017–2.04 nM (Figure 3B–F). There was no IFN-gamma secretion by BCMA-CD3 antibodies with T cells and BCMA-negative CHO cells (Figure S1B). Thus, all BCMA-CD3 antibodies had high efficacy with CHO-BCMA target cells.

### 3.4. BCMA-CD3 Antibodies Bind to BCMA-Positive Multiple Myeloma Cells and Cause Secretion of IFN-Gamma

**Figure 4:**
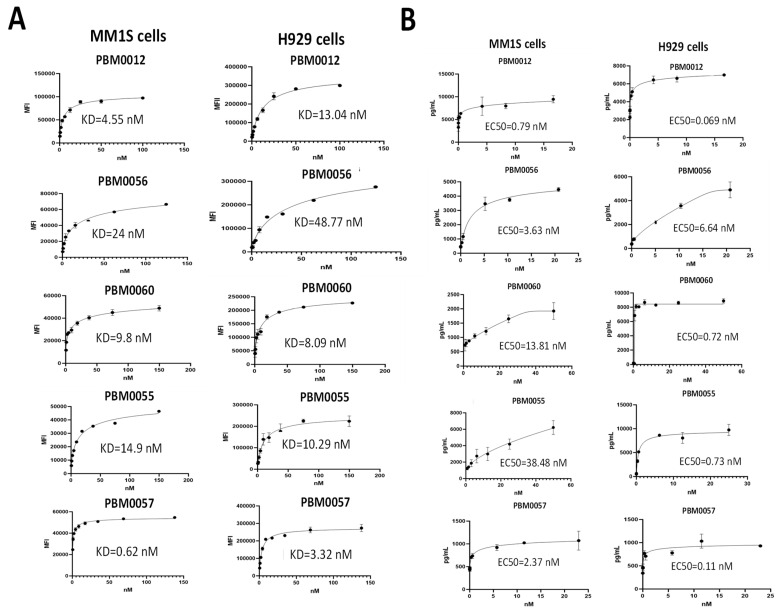
(**A**) BCMA-CD3 antibodies bind multiple myeloma cell lines and all bispecific antibodies with T cells secrete IFN-gamma. (**A**) Representative FACS assay with MM1S and H929 cells is shown. (**B**) BCMA-CD3 antibodies and T cells secrete IFN-gamma with multiple myeloma cells. Representative assay is shown. Bars show standard deviations from three independent measurements.

BCMA-CD3 antibodies were tested for binding with MM1S and H929 multiple myeloma cell lines. FACS analysis with BCMA-CD3 antibodies demonstrated that all antibodies bound BCMA-positive multiple myeloma cell lines with KD in the nM range (Figure 4A). There was no binding of BCMA-CD3 antibodies to BCMA-negative lymphablstoid K562 cell line, while there was positive binding to RPMI8226 multiple myeloma cell line (Figure S2A).

We tested these antibodies for secretion of INF-gamma with multiple myeloma cell lines (Figure 4B). All antibodies secreted high levels of IFN-gamma and EC50 was from 0.7–38 nM. There was no high secretion of IFN-gamma by T cells and BCMA-CD3 antibodies in the BCMA-negative K562 cell line (Figure S2B) and in BCMA-negative primary B cells (Figure S3). Thus, BCMA-CD3 antibodies effectively and specifically bound BCMA-positive multiple myeloma cell lines and all antibodies with T cells secreted IFN-gamma.

### 3.5. BCMA-CD3 Antibodies with T Cells Effectively Block Multiple Myeloma Tumor Growth In Vivo

**Figure 5:**
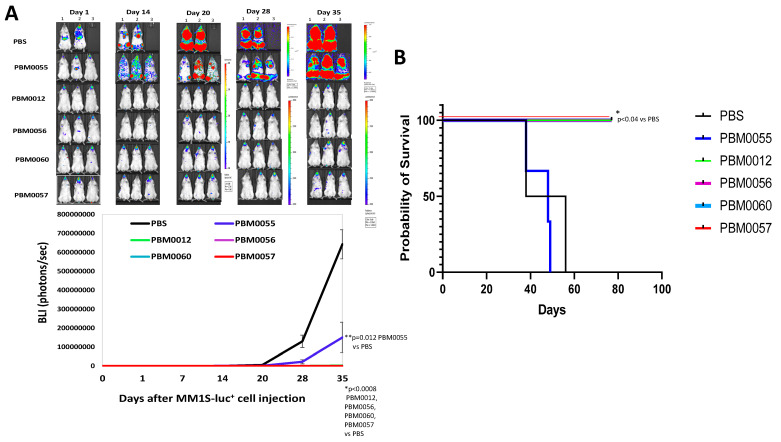
BCMA-CD3 antibodies with T cells significantly decrease MM1S-luciferase+ tumor growth. (**A**) Imaging of MM1S-luc xenografts with BCMA-CD3 antibodies and T cells. The PBS-treated group had two mice; all BCMA-CD3-treated groups had three mice per group (*n* = 3). Upper panel: Images of mice. Lower panel Quantification of BLI (photons/sec). *p* < 0.0008, BLI for PBM0012, PBM0056, PBM0060 and PBM0057 versus PBS group. *p* = 0.012, BLI for PBM0055 versus PBS-group, Student’s *t*-test. (**B**) Kaplan Myer survival curve. *p* < 0.04 all antibodies vs. PBS control by Log-rank Mantel-Cox test.

We tested the efficacy of BCMA-CD3 antibodies using the MM1S xenograft mouse NSG model (Figure 5). MM1S-luciferase-positive cells were injected intravenously into mice and antibodies with T cells were injected intravenously three times. All antibodies except PBM0055 significantly decreased tumor growth with *p* < 0.001 ((Figure 5A). In addition, PBM0012, PBM0056, PBM0060, and PBM0057 antibodies with T cells significantly prolonged mouse survival (*p* < 0.04) (Figure 5B). Thus, three bispecific BCMA-CD3 antibodies significantly decreased MM1S tumor growth similarly to the control PBM0057 antibody.

In addition, we performed an additional mouse in vivo efficacy study using CAR-T cells with humanized BCMA Scfv with the same VH and VL as in BCMA-CD3 antibodies (Figure S4). We designed humanized BCMA ScFv-41BB-CD3 CAR shown in Figure S4A. The generated hBCMA-CAR-T cells expressed more than 70% BCMA-CAR-positive cells (Figure S4B). The CAR-T cells killed CHO-BCMA cells and did not kill CHO cells (Figure S4C) and secreted a high level of IFN-gamma with CHO-BCMA target cells (Figure S4D). Moreover, hBCMA-CAR-T cells significantly (*p* < 0.05) decreased RPMI8226-luciferase^+^ multiple myeloma xenograft tumor growth by imaging (Figure S4E) and significantly prolonged mouse survival *p* < 0.02 (Figure S4E). The high in vivo efficacy of the humanized BCMA-CAR-T cells in the multiple myeloma xenograft model confirms the high in vivo efficacy of BCMA-CD3 bispecific antibodies.

## 4. Discussion

This report demonstrated the generation of several antibody designs bivalent, univalent BCMA-CD3 Crossmab KIH; and BCMA-CD3 ScFv KIH with two different CD3 ScFv humanized sequences. We tested these antibodies for binding with BCMA-positive cell lines and detected that all antibodies specifically bound with high KD to BCMA-positive CHO-BCMA cells. In addition, they all bound CD3-positive T cells. All antibodies also bound multiple myeloma cell lines with KD in the nM range. BCMA-CD3 antibodies did not bind CHO, K562, and primary B cells. The BCMA-CD3 antibodies with T cells killed BCMA-positive CHO-BCMA cell lines and secreted IFN-gamma with EC50 in the nM range. BCMA-CD3 with T cells did not secrete IFN-gamma with BCMA-negative cells. Moreover, three BCMA-CD3 antibodies significantly decreased MM1S-luc^+^ xenograft tumor growth and prolonged mouse survival in vivo except for one BCMA-CD3 antibody PBM0055 which has different CD3 ScFv. Thus, this report demonstrates the high in vitro and in vivo efficacy of novel BCMA-CD3 bispecific antibodies with different designs. 

In addition, we confirmed the high in vitro and in vivo efficacy of BCMA-CD3 antibodies with humanized BCMA-CAR-T cells containing the same humanized BCMA ScFv (Figure S4). Future pre-clinical studies will be performed to compare both types of therapies. 

The BCMA-CD3 antibodies bound CHO-BCMA cells and did not bind CHO and K562 cells. The bivalent CrossMab and univalent CrossMab KIH bound BCMA-positive cells with similar KD with both CHO-BCMA and with multiple myeloma cell lines. These antibodies had lower binding to T cells than other designs suggesting that designs with CD3 ScFv have higher binding to T cells. PBM0055 did not have in vivo activity that can be explained by different humanized CD3 ScFv compared to other designs. The future study will detect the differences between humanized CD3 sequences and the effect of several amino-acid changes on antibody functions in vivo. 

The future pre-clinical efficacy, toxicology, and pharmacokinetics studies will be performed with different doses of antibodies, larger mouse groups, and humanized mouse models to reveal differences between different designs of antibodies. This is the first study to demonstrate in vitro and in vivo functional efficacy of these novel BCMA-CD3 antibodies. 

Several BCMA-CD3 antibodies were tested in clinical studies in different formats: AMG420, BITE BCMA-CD3, and AMG701, half-life extended HLE-BITE by Amgen; CC-93269, EM901 CrossMab KIH bivalent BCMA-CD3 antibody by Celgene; PF-06863135 BCMA-CD3 antibody by Pfizer; JNJ-64007957, Duobody by Janssen and REGN5458, hetero H, CL IgG4 with Fc silenced by Regeneron [24]. The AMG420 BITE BCMA-CD3 antibody had a short half-life and needed to be administered for 4 weeks, while HLE-BITE AMG701 required to be administered only once a week and now it is tested in phase I clinical trial [24]. EM-801 CrossMAB bivalent KIH BCMA-CD3 bispecific antibody has an advantage of prolonged stability and can be administered intravenously weekly [25]. The first clinical result of the related EM901 antibody showed that more than 90 percent of patients responded to the treatment [24]. Pfizer’s PF-06863135 antibody showed high in vitro and in vivo efficacy [26] and is now tested in a clinical phase I trial (NCT03269136) [24]. Each of the mentioned antibodies has its own mechanism and its own advantages and disadvantages [24]. We presented novel BCMA-CD3 antibodies with high in vitro and in vivo activities. More mechanistic, efficacy and toxicology pre-clinical studies will be performed in a future report to compare present antibodies to other BCMA-CD3 antibodies. There are several challenges like cytokine release syndrome, inhibitory checkpoint signaling, inhibitory tumor microenvironment, and other factors that need to be addressed in future preclinical and clinical studies [24]. Novel bispecific BCMA-CD3 antibodies are important to develop for better therapeutic targeting of multiple myeloma.

In summary, the presented novel BCMA-CD3 antibodies with different designs containing CD3 sequences of PBM0012, PBM0056, and PBM0060 have high in vitro and in vivo activity. 

## 5. Conclusions

This study demonstrates the high efficacy of novel BCMA-CD3 antibodies in vitro and in vivo. The data provide a basis for future pre-clinical and clinical studies.

## 6. Patents

Humanized BCMA VH, VL, and Scfv and bispecific antibody sequences are included in the Promab Biotechnology patent application.

## Data Availability

Data are contained within the article.

## Supplementary Materials

The following supporting information can be downloaded at: https://www.mdpi.com/article/10.3390/cancers14102518/s1. Table S1, Amino-acid sequence of BCMA-CD3 antibodies used in the study; Figure S1, A. FACS with BCMA-CD3 antibodies shows no binding with BCMA-negative CHO cells. B. IFN-gamma ELISA assay shows no secretion of IFN-gamma by T cells with BCMA-CD3 antibodies with CHO target cells; Figure S2, A. FACS with BCMMA-CD3 antibodies shows no binding of BCMA-CD3 antibodies to K562 lymphoblast cells. B. T cells with BCMA-CD3 antibodies don’t secrete high level IFN-gamma with K562 cells; Figure S3, T cells with BCMA-CD3 antibodies secrete significantly less IFN-gamma with B cells than with MM1S multiple myeloma cells; Figure S4, Humanized BCMA-CAR-T cells significantly block RPMI8226-luciferase+ multiple myeloma xenograft tumor growth.
