# Supplementary material for: Bispecific BCMA-CD3 Antibodies Block Multiple Myeloma Tumor Growth
Source: Cancers (Basel). 2022 May 20;14(10):2518. doi: 10.3390/cancers14102518 (PMC9139578; doi:10.3390/cancers14102518)
Supplement: Supplementary file 1 [file cancers-14-02518-s001.zip › cancers-1687441-supplementary.pdf]

Supplementary Material

Table S1. Amino-acid sequence of BCMA-CD3 antibodies used in the study.

	PBM0012
Subunit 1	DVVMTQSPAFLSVTPGEKVTITCRASQSISDYLHWYQQKPDQAPKLLIKYASQSISGVPSRFSGS<br>GSGTDFTFTISSLEAEDAATYYCQNGHSFPPTFGGGTKVEIKRTVAAPSVFIFPPSDEQLKSGTAS<br>VVCLLNNFYPREAKVQWKVDNALQSGNSQESVTEQDSKDSTYSLSSTLTLSKADYEKHKVYA<br>CEVTHQGLSSPVTKSFNRGEC
Subunit 2	Q A V V T Q E P S L T V S P G G T V T L T C G S S T G A V T T S N Y A N W V Q E K P G Q A F<br>R G L I G G T N K R A P G T P A R F S G S L L G G K A A L T L S G A Q P E D E A E Y Y C A L<br>W Y S N L W V F G G G T K L T V L S S A S T K G P S V F P L A P S S K S T S G G T A A L G C<br>L V K D Y F P E P V T V S W N S G A L T S G V H T F P A V L Q S S G L Y S L S S V V T V P S S<br>S L G T Q T Y I C N V N H K P S N T K V D K K V E P K S C
Subunit 3	QVQLVQSGAEVKKPGSSVKVSCKASGYTFTSYVMHWVRQAPGQGLEWMGYIIPYNDATKYN<br>EKFKGRVTITADKSTSTAYMELSSLRSEDTAVYYCARYNYDGYFDVWGQGTLVTVSSTKGPSV<br>FPLAPSSKSTSGGTAALGCLVKDYFPEPVTVSWNSGALTSGVHTFPAVLQSSGLYSLSSVVTVPS<br>SSLGTQTYICNVNHKPSNTKVDKKVEPKSCDGGGGSGGGGSEVQLLESGGGLVQPGGSLRLS<br>CAASGFTFSTYAMNWVRQAPGKGLEWVSRIRSKYNNYATYYADSVKGRFTISRDDSKNTLYL<br>QMNSLRAEDTAVYYCVRHGNFGNSYVSWFAYWGQGTLVTVSSASVAAPSVFIFPPSDEQLKS<br>GTASVVCLLNNFYPREAKVQWKVDNALQSGNSQESVTEQDSKDSTYSLSSTLTLSKADYEKH<br>KVYACEVTHQGLSSPVTKSFNRGECDKTHTCPPCPAPEAAGGPSVFLFPPKPKDTLMISRTPEV<br>TCVVVDVSHEDPEVKFNWYVDGVEVHNAKTKPREEQYNSTYRVVSVLTVLHQDWLNGKEY<br>KCKVSNKALGAPIEKTISKAKGQPREPQVYTLPPCRDELTKNQVSLWCLVKGFYPSDIAVEWE<br>SNGQPENNYKTTPPVLDSDGSFFLYSKLTVDKSRWQQGNVFSCSVMHEALHNHYTQKSLSLS<br>PGK
Subunit 4	QVQLVQSGAEVKKPGSSVKVSCKASGYTFTSYVMHWVRQAPGQGLEWMGYIIPYNDATKYN<br>EKFKGRVTITADKSTSTAYMELSSLRSEDTAVYYCARYNYDGYFDVWGQGTLVTVSSTKGPSV<br>FPLAPSSKSTSGGTAALGCLVKDYFPEPVTVSWNSGALTSGVHTFPAVLQSSGLYSLSSVVTVPS<br>SSLGTQTYICNVNHKPSNTKVDKKVEPKSCDKTHTCPPCPAPEAAGGPSVFLFPPKPKDTLMIS<br>RTPEVTCVVVDVSHEDPEVKFNWYVDGVEVHNAKTKPREEQYNSTYRVVSVLTVLHQDWL<br>NGKEYKCKVSNKALGAPIEKTISKAKGQPREPQV*C*TLPPSRDELTKNQVSL*S*C*A*VKGFYPSDIA<br>VEWESNGQPENNYKTTPPVLDSDGSFFL*V*SKLTVDKSRWQQGNVFSCSVMHEALHNHYTQK<br>SLSLSPGK
	PBM0056
Subunit 1	DVVMTQSPAFLSVTPGEKVTITCRASQSISDYLHWYQQKPDQAPKLLIKYASQSISGVPSRFSGS<br>GSGTDFTFTISSLEAEDAATYYCQNGHSFPPTFGGGTKVEIKRTVAAPSVFIFPPSDEQLKSGTAS<br>VVCLLNNFYPREAKVQWKVDNALQSGNSQESVTEQDSKDSTYSLSSTLTLSKADYEKHKVYA<br>CEVTHQGLSSPVTKSFNRGEC
Subunit 2	Q A V V T Q E P S L T V S P G G T V T L T C G S S T G A V T T S N Y A N W V Q E K P G Q A F<br>R G L I G G T N K R A P G T P A R F S G S L L G G K A A L T L S G A Q P E D E A E Y Y C A L<br>W Y S N L W V F G G G T K L T V L S S A S T K G P S V F P L A P S S K S T S G G T A A L G C<br>L V K D Y F P E P V T V S W N S G A L T S G V H T F P A V L Q S S G L Y S L S S V V T V P S S<br>S L G T Q T Y I C N V N H K P S N T K V D K K V E P K S C
Subunit 3	EVQLLESGGGLVQPGGSLRLSCAASGFTFSTYAMNWVRQAPGKGLEWVSRIRSKYNNYATYY<br>ADSVKGRFTISRDDSKNTLYLQMNSLRAEDTAVYYCVRHGNFGNSYVSWFAYWGQGTLVTV<br>SSASVAAPSVFIFPPSDEQLKSGTASVVCLLNNFYPREAKVQWKVDNALQSGNSQESVTEQDS

	KDSTYSLSSTLTLSKADYEKHKVYACEVTHQGLSSPVTKSFNRGECDKTHTCPPCPAPEAAGG<br>PSVFLFPPKPKDTLMISRTPEVTCVVVDVSHEDPEVKFNWYVDGVEVHNAKTKPREEQYNSTY<br>RVVSVLTVLHQDWLNGKEYKCKVSNKALGAPIEKTISKAKGQPREPQVYTLPPCRDELTKNQ<br>VSLWCLVKGFYPSDIAVEWESNGQPENNYKTTPPVLDSDGSFFLYSKLTVDKSRWQQGNVFS<br>CSVMHEALHNHYTQKSLSLSPGK
Subunit 4	QVQLVQSGAEVKKPGSSVKVSCKASGYTFTSYVMHWVRQAPGQGLEWMGYIIPYNDATKYN<br>EKFKGRVTITADKSTSTAYMELSSLRSEDTAVYYCARYNYDGYFDVWGQGTLVTVSSTKGPSV<br>FPLAPSSKSTSGGTAALGCLVKDYFPEPVTVSWNSGALTSGVHTFPAVLQSSGLYSLSSVVTVPS<br>SSLGTQTYICNVNHKPSNTKVDKKVEPKSCDKTHTCPPCPAPEAAGGPSVFLFPPKPKDTLMIS<br>RTPEVTCVVVDVSHEDPEVKFNWYVDGVEVHNAKTKPREEQYNSTYRVVSVLTVLHQDWL<br>NGKEYKCKVSNKALGAPIEKTISKAKGQPREPQV*C*TLPPSRDELTKNQVSL*SCA*VKGFYPSDIA<br>VEWESNGQPENNYKTTPPVLDSDGSFFL*V*SKLTVDKSRWQQGNVFSCSVMHEALHNHYTQK<br>SLSLSPGK
	PBM0060
Subunit 1	QVQLVQSGAEVKKPGSSVKVSCKASGYTFTSYVMHWVRQAPGQGLEWMGYIIPYNDATKYN<br>EKFKGRVTITADKSTSTAYMELSSLRSEDTAVYYCARYNYDGYFDVWGQGTLVTVSSASTKGP<br>SVFPLAPSSKSTSGGTAALGCLVKDYFPEPVTVSWNSGALTSGVHTFPAVLQSSGLYSLSSVVTV<br>PSSSLGTQTYICNVNHKPSNTKVDKKVEPKSCDKTHTCPPCPAPEAAGGPSVFLFPPKPKDTL<br>MISRTPEVTCVVVDVSHEDPEVKFNWYVDGVEVHNAKTKPREEQYNSTYRVVSVLTVLHQD<br>WLNGKEYKCKVSNKALPAPIEKTISKAKGQPREPQVYTLPP*C*RDELTKNQVSL*W*CLVKGFYPS<br>DIAVEWESNGQPENNYKTTPPVLDSDGSFFLYSKLTVDKSRWQQGNVFSCSVMHEALHNHY<br>TQKSLSLSPGK
Subunit 2	EVQLLESGGGLVQPGGSLRLSCAASGFTFSTYAMNWVRQAPGKGLEWVSRIRSKYNNYATYY<br>ADSVKGRFTISRDDSKNTLYLQMNSLRAEDTAVYYCVRHGNFGNSYVSWFAYWGQGTLVTV<br>SSGGGGSGGGGSGGGGSQAVVTQEPSLTVSPGGTVTLTCGSSTGAVTTSNYANWVQEKPGQA<br>FRGLIGGTNKRAPGTPARFSGSLLGGKAALTLSGAQPEDEAEYYCALWYSNLWVFGGGTKLT<br>VLGGGGSGGGGSGGGGSGGGGSGGGGSAAATHTCPPCPAPEAAGGPSVFLFPPKPKDTLMIS<br>RTPEVTCVVVDVSHEDPEVKFNWYVDGVEVHNAKTKPREEQYNSTYRVVSVLTVLHQDWL<br>NGKEYKCKVSNKALPAPIEKTISKAKGQPREPQVYTLPPSRDELTKNQVSLSCAVKGFYPSDIA<br>VEWESNGQPENNYKTTPPVLDSDGSFFLVSKLTVDKSRWQQGNVFSCSVMHEALHNHYTQK<br>SLSLSPGK
Subunit 3	DVVMTQSPAFLSVTPGEKVTITCRASQSISDYLHWYQQKPDQAPKLLIKYASQSISGVPSRFSGS<br>GSGTDFTFTISSLEAEDAATYYCQNGHSFPPTFGGGTKVEIKRTVAAPSVFIFPPSDEQLKSGTAS<br>VVCLLNNFYPREAKVQWKVDNALQSGNSQESVTEQDSKDSTYSLSSTLTLSKADYEKHKVYA<br>CEVTHQGLSSPVTKSFNRGEC
	PBM0055
Subunit 1	QVQLVQSGAEVKKPGSSVKVSCKASGYTFTSYVMHWVRQAPGQGLEWMGYIIPYNDATKYN<br>EKFKGRVTITADKSTSTAYMELSSLRSEDTAVYYCARYNYDGYFDVWGQGTLVTVSSASTKGP<br>SVFPLAPSSKSTSGGTAALGCLVKDYFPEPVTVSWNSGALTSGVHTFPAVLQSSGLYSLSSVVTV<br>PSSSLGTQTYICNVNHKPSNTKVDKKVEPKSCDKTHTCPPCPAPEAAGGPSVFLFPPKPKDTL<br>MISRTPEVTCVVVDVSHEDPEVKFNWYVDGVEVHNAKTKPREEQYNSTYRVVSVLTVLHQD

	WLNGKEYKCKVSNKALPAPIEKTISKAKGQPREPQVYTLPPCRDELTKNQVSLWCLVKGFYPS<br>DIAVEWESNGQPENNYKTTPPVLDSDGSFFLYSKLTVDKSRWQQGNVFSCSVMHEALHNHY<br>TQKSLSLSPGK
Subunit 2	EVQLVESGGGLVQPGGSLKLSCAASGFTFNKYAMNWVRQAPGKGLEWVARIRSKYNNYATY<br>YADSVKDRFTISRDDSKNTAYLQMNNLKTEDTAVYYCVRHGNFGNSYISYWAYWGQGTLVT<br>VSSGGGGSGGGGSGGGGSQTVVTQEPSLTVSPGGTVTLTCGSSTGAVTSGNYPNWVQQKPGQ<br>APRGLIGGTKFLAPGTPARFSGSLLGGKAALTLSGVQPEDEAEYYCVLWYSNRWVFGGGTKL<br>TVLGGGGSGGGGSGGGGSGGGGSGGGGSAAATHTCPPCPAPEAAGGPSVFLFPPKPKDTLMI<br>SRTPEVTCVVVDVSHEDPEVKFNWYVDGVEVHNAKTKPREEQYNSTYRVVSVLTVLHQDWL<br>NGKEYKCKVSNKALPAPIEKTISKAKGQPREPQVYTLPPSRDELTKNQVSLSCAVKGFYPSDIA<br>VEWESNGQPENNYKTTPPVLDSDGSFFLVSKLTVDKSRWQQGNVFSCSVMHEALHNHYTQK<br>SLSLSPGK
Subunit 3	DVVMTQSPAFLSVTPGEKVTITCRASQSISDYLHWYQQKPDQAPKLLIKYASQSISGVPSRFSGS<br>GSGTDFTFTISSLEAEDAATYYCQNGHSFPPTFGGGTKVEIKRTVAAPSVFIFPPSDEQLKSGTAS<br>VVCLLNNFYPREAKVQWKVDNALQSGNSQESVTEQDSKDSTYSLSSTLTLSKADYEKHKVYA<br>CEVTHQGLSSPVTKSFNRGEC
	PBM0057
Subunit 1	E V Q L V E S G G G L V Q P G G S L R L S C A A S G F T F S D Y Y M T W V R Q A P G K G L<br>E W V A F<br>I R N R A R G Y T S D H N P S V K G R F T I S R D N A K N S L Y L Q M N S L R A E D T A V Y<br>Y C A R<br>D R P S Y Y V L D Y W G Q G T T V T V S S A S T K G P S V F P L A P C S R S T S E S T A A L G<br>C L V<br>K D Y F P E P V T V S W N S G A L T S G V H T F P A V L Q S S G L Y S L S S V V T V P S S N F<br>G T Q<br>T Y T C N V D H K P S N T K V D K T V E R K C R V R C P R C P A P P V A G P S V F L F P P K<br>P K D T<br>L M I S R T P E V T C V V V A V S H E D P E V Q F N W Y V D G V E V H N A K T K P R E E Q<br>F N S T F<br>R V V S V L T V V H Q D W L N G K E Y K C K V S N K G L P S S I E K T I S K T K G Q P R E P<br>Q V Y T<br>L P P S R E E M T K N Q V S L T C L V K G F Y P S D I A V E W E S N G Q P E N N Y K T T P P<br>M L D S<br>D G S F F L Y S R L T V D K S R W Q Q G N V F S C S V M H E A L H N H Y T Q K S L S L S P G<br>K
Subunit 2	E V Q L L E S G G G L V Q P G G S L R L S C A A S G F T F S S Y P M S W V R Q A P G K G L E<br>W V S A<br>I G G S G G S L P Y A D I V K G R F T I S R D N S K N T L Y L Q M N S L R A E D T A V Y Y C<br>A R Y W<br>P M D I W G Q G T L V T V S S A S T K G P S V F P L A P C S R S T S E S T A A L G C L V K D<br>Y F P E<br>P V T V S W N S G A L T S G V H T F P A V L Q S S G L Y S L S S V V T V P S S N F G T Q T Y T<br>C N V<br>D H K P S N T K V D K T V E R K C E V E C P E C P A P P V A G P S V F L F P P K P K D T L M<br>I S R T<br>P E V T C V V V A V S H E D P E V Q F N W Y V D G V E V H N A K T K P R E E Q F N S T F R<br>V V S V L<br>T V V H Q D W L N G K E Y K C K V S N K G L P S S I E K T I S K T K G Q P R E P Q V Y T L P<br>P S R E

	E M T K N Q V S L T C E V K G F Y P S D I A V E W E S N G Q P E N N Y K T T P P M L D S D G S F F L<br>Y S K L T V D K S R W Q Q G N V F S C S V M H E A L H N H Y T Q K S L S L S P G K
Subunit 3	D I V M T Q S P D S L A V S L G E R A T I N C K S S Q S L F N V R S R K N Y L A W Y Q Q K P G Q P P<br>K L L I S W A S T R E S G V P D R F S G S G S G T D F T L T I S S L Q A E D V A V Y Y C K Q S Y D L<br>F T F G S G T K L E I K R T V A A P S V F I F P P S D E Q L K S G T A S V V C L L N N F Y P R E A K V Q W K V D N A L Q S G N S Q E S V T E Q D S K D S T Y S L S S T L T L S K A D Y E K H K V Y A C E V T H Q G L S S P V T K S F N R G E C
Subunit 4	E I V L T Q S P G T L S L S P G E R A T L S C R A S Q S V S S S Y L A W Y Q Q K P G Q A P R L L M Y<br>D A S I R A T G I P D R F S G S G S G T D F T L T I S R L E P E D F A V Y Y C Q Q Y Q S W P L T F G<br>Q G T K V E I K R T V A A P S V F I F P P S D E Q L K S G T A S V V C L L N N F Y P R E A K V Q W K<br>V D N A L Q S G N S Q E S V T E Q D S K D S T Y S L S S T L T L S K A D Y E K H K V Y A C E V T H Q<br>G L S S P V T K S F N R G E C

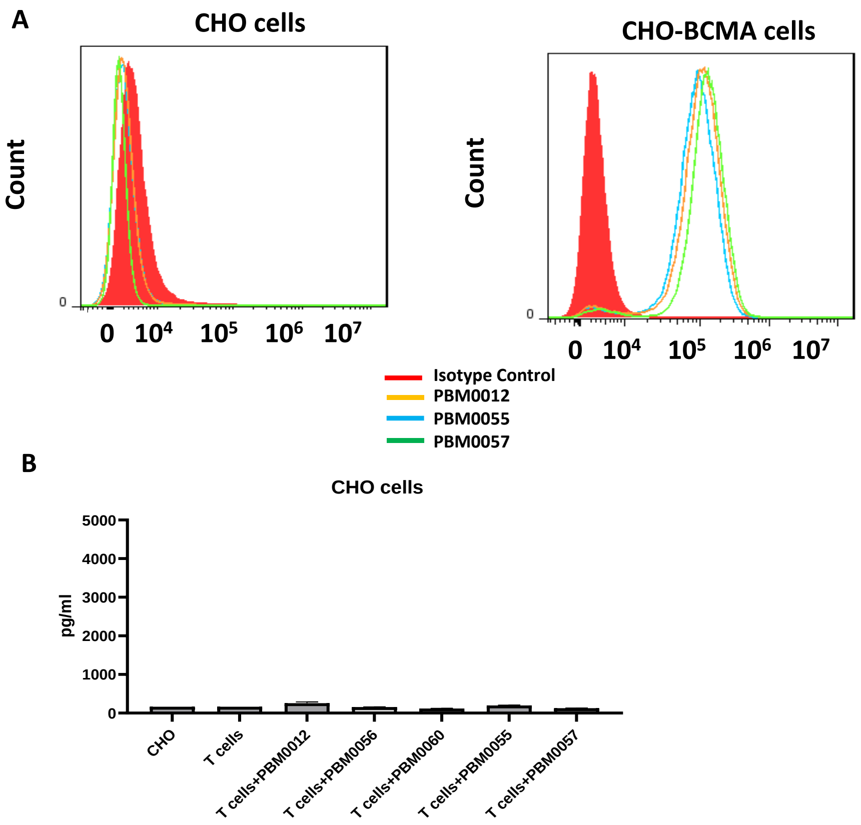

Figure S1. (**A**) FACS with BCMA-CD3 antibodies shows no binding with BCMA-negative CHO cells. CHO-BCMA cells show positive binding with BCMA-CD3 antibodies. Left panel FACS with CHO cells, right panel FACS with CHO-BCMA cells. Representative FACS with PBM0012, PBM0055 and PBM0057 BCMA-CD3 antibodies is shown. (**B**) IFN-gamma ELISA assay as described in Materials and Methods shows no secretion of IFN-gamma by T cells with BCMA-CD3 antibodies with CHO target cells. Bars show average ± standard deviations.

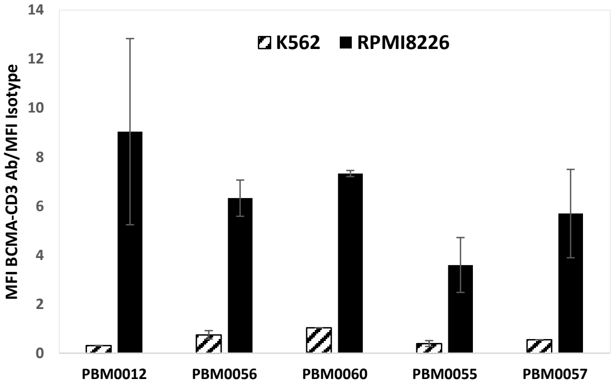

(A)

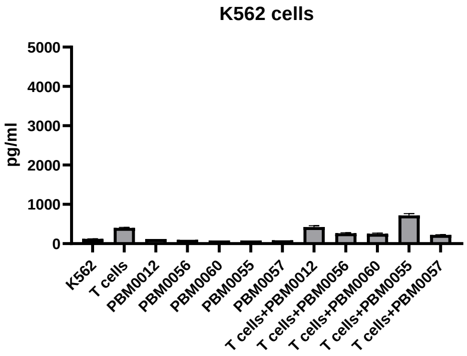

(B)

Figure S2. (**A**) FACS with BCMMA-CD3 antibodies shows no binding of BCMA-CD3 antibodies to K562 lymphoblast cells. Multiple myeloma RPMI8226 cells show positive binding with BCMA-CD3 antibodies. MFI BCMA Abs/MFI isotype antibody is shown on Y-axis. (**B**) T cells with BCMA-CD3 antibodies don't secrete high level IFN-gamma with K562 cells. IFN-gamma ELISA assay was performed as described in Materials and Methods. Bars show average ± standard deviations.

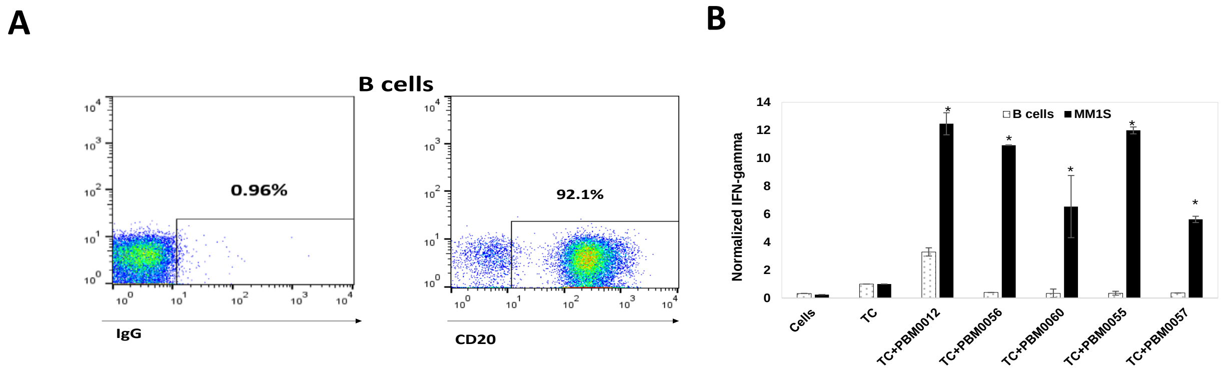

Figure S3. T cells with BCMA-CD3 antibodies secrete significantly less IFN-gamma with B cells than with MM1S multiple myeloma cells. (**A**) FACS with B cell marker, CD2O antibody shows >90% CD20-positive cells in expanded primary B cells. The binding was negative with BCMA-CD3 antibody (not shown). (**B**) Low level of IFN-gamma secreted by T cells and BCMA-CD3 antibodies with primary B cells. Bars show average ± standard deviations. IFN-gamma is normalized to the level of IFN-gamma secreted by T cells (TC) alone with target cells. * $p < 0.05$, IFN-gamma secreted by BCMA-CD3 antibodies with T cells with multiple myeloma MM1S target cells versus INF-gamma secreted with target B cells.

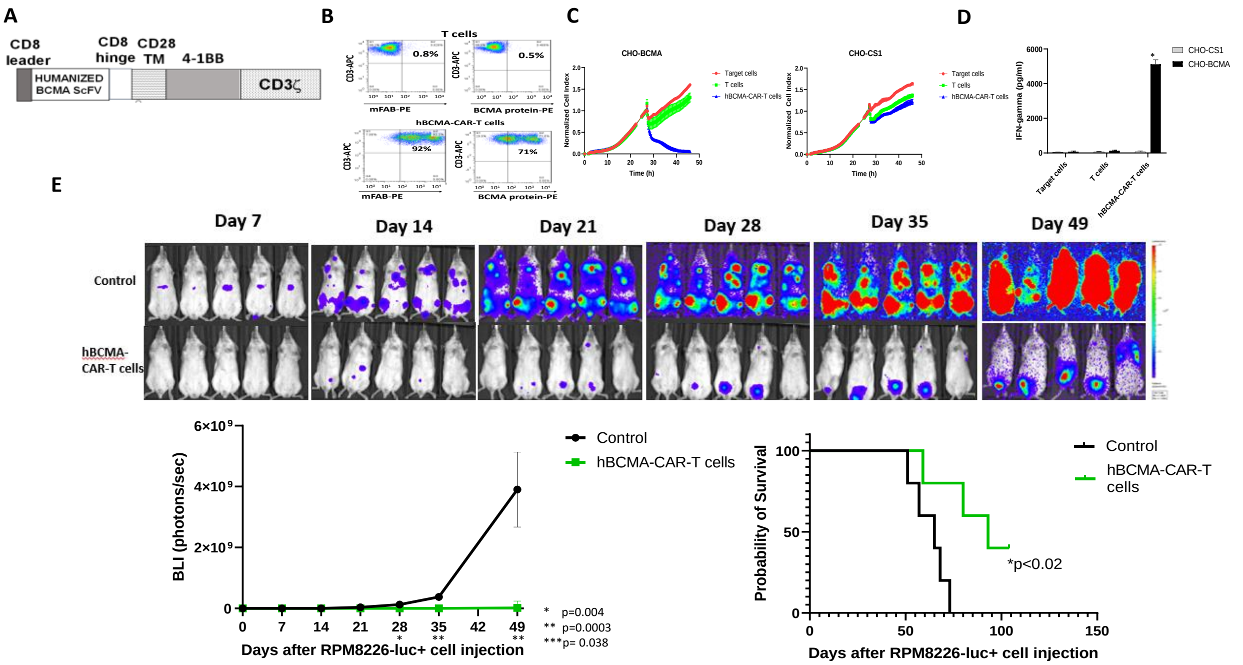

Figure S4. Humanized BCMA-CAR-T cells significantly block RPMI8226-luciferase+ multiple myeloma xenograft tumor growth. (**A**) The structure of humanized hBCMA-CAR construct. BCMA ScFv contains VH and VL (Table S1) linked with G4S linker. CAR structure contains CD8 alpha hinge, CD28 transmembrane, 4-1BB co-stimulatory and CD3 activation domains. (**B**) FACS detects CAR-positive T cells after transduction of T cells with hBCMA-CAR lentivirus. FACS was performed on BCMA-CAR-T cells with anti-mouse-F(ab)$_2$ antibody (left panel) and with recombinant BCMA protein (right panel). (**C**) hBCMA-CAR-T cells kill CHO-BCMA cells (left) and don't kill CHO-CS1 target cells (right). RTCA assay was performed as described in Materials and Methods. (**D**) hBCMA-CAR-T cells secrete high level of IFN-gamma with CHO-BCMA cells. $p < 0.0004$, Student's t-test. IFN-gamma secreted by hBCMA-CAR-T cells with CHO-BCMA cells compared with CHO-CS1 target cells. (**E**) hBCMA-CD3 CAR-T cells significantly decreased RPMI8226-luc$^+$ xenograft tumor growth. Upper panel shows imaging of BCMA-CAR-T cell-treated mice (n = 5 mice/group). Lower left panel shows quantification of imaging BLI (photons/sec). * $p < 0.004$, BLI of BCMA-treated mice at day 28; ** $p < 0.0003$ at day 35; *** $p < 0.04$ at day 49 versus control group by Student's t-test. Lower right panel. BCMA-CAR-T cells significantly prolong survival of RPMI8226 xenograft NSG mice. * $p < 0.02$ of BCMA-CAR-T cell treated versus control mice by Log-rank Mantel-Cox test.
